# ARCH: IMPACTING RESEARCH, IMPACTING CAREERS

**DOI:** 10.1093/geroni/igae098.1548

**Published:** 2024-12-31

**Authors:** Victoria Helmly, Jennifer James

**Affiliations:** Georgia State University, Atlanta, Georgia, United States; University of California San Francisco, San Francisco, California, United States

## Abstract

Despite more than 50 years of unprecedented growth in incarcerated populations and the rapidly increasing age of people in prisons and jails, the amount of research and the number of researchers focused on aging and the criminal legal system remains small. For many early career scholars interested in aging, health, and incarceration, supportive mentorship, community, collaboration, and funding opportunities have remained elusive. It is common for scholars studying aging within the criminal legal system to be the only researchers in their institution focused on this critical area. The Aging Research in Criminal Justice Health (ARCH) Network seeks to address this gap by fostering collaboration among researchers and experts in this crucial intersection while providing essential mentorship and support for early career development. Over the last five years, ARCH has created opportunities for Ph.D. students and early-career researchers to contribute to research projects, co-author manuscripts, build networks, hone their skills, and receive feedback on their work. In this session, presenters will share their experience in the ARCH network and how it has impacted their careers through connections and collaborations with mentors and peers. They will describe the ARCH network as a pipeline model for advancing scholars interested in health equity and policy-focused research.

